# The potential use of chickpeas in development of infant follow-on formula

**DOI:** 10.1186/1475-2891-13-8

**Published:** 2014-01-22

**Authors:** Lovemore Nkhata Malunga, Shimrit Bar-El Dadon, Eli Zinal, Zipi Berkovich, Shahal Abbo, Ram Reifen

**Affiliations:** 1 The School of Nutritional Sciences Plant Genetics, The Hebrew University of Jerusalem, P.O. Box 12, Rehovot 76100, Israel; 2 Plant Genetics The Hebrew University of Jerusalem, P.O. Box 12, Rehovot 76100, Israel; 3 Tnuva Research and Development Center, Rehovot, Israel

**Keywords:** Chickpea, Protein, Weaning formulae, Amino acid, Infant follow-on formulae

## Abstract

**Background:**

Undernutrition during childhood is a common disorder in the developing countries, however most research has focussed much on its treatment rather than its prevention.

**Objective:**

We investigated the potential of using chickpeas in infant follow-on formula production against the requirements of WHO/FAO on complementary foods and EU regulations on follow-on formula.

**Methods:**

Chickpeas were germinated for 72 hours followed by boiling, drying and dehulling in order to minimise associated anti-nutrition factors. Saccharifying enzymes were used to hydrolyse starch to maltose and the resulting flours were analysed for their protein content and amino acid profile.

**Results:**

The protein content (percentage) increased from 16.66 ± 0.35 and 20.24 ± 0.50 to 20.00 ± 0.15 and 21.98 ± 0.80 for the processed desi and kabuli cultivar compared to raw chickpeas, respectively (P < 0.05). There was insignificant change (P = 0.05) in amino acid profile following processing and the resulting flour was found to meet the amino acid requirements of WHO/FAO protein reference for 0–24 month’s children.

**Conclusion:**

The designed chickpea based infant follow-on formula meets the WHO/FAO requirements on complementary foods and also the EU regulations on follow-on formula with minimal addition of oils, minerals and vitamins. It uses chickpea as a common source of carbohydrate and protein hence making it more economical and affordable for the developing countries without compromising the nutrition quality.

## Introduction

Childhood malnutrition caused by the consumption of low nutrient density of weaning foods is common in the developing countries [1]. The growth rate among children of developing and developed countries is not significantly different during the first 4–6 months of life, a period characterised by breastfeeding [2,3]. However, the growth rate falters in children in developing countries immediately after the introduction of weaning foods which are usually of poor nutritional value [1-3]. Hence, nutritionally adequate and safe complementary feeding starting from the age of 6 months with continued breastfeeding up to 24 months of age or beyond is recommended by the World Health Organisation (WHO).

The use of novel follow on formulas, common in the developed world, to prevent early childhood malnutrition has not been tried in the developing world as research has focussed mainly on treatment rather than prevention [4]. The major concern to introduce infant follow on formula has been that of affordability and hygiene. Therefore, the introduction of a cost effective novel infants follow on (weaning) formula using simple, affordable, and sustainable technologies will likely improve the overall children nutrition in the developing countries.

Conventionally, infant follow on formulas in the developing world are exclusively made from either cow’s milk protein or soy protein isolate blended with starch or corn syrup or dextrose, vegetable oil/fat and mineral and vitamin premixes [5]. In many developing countries, commercially available weaning foods are too expensive for the average family, so nursing mothers often depend on traditional weaning foods which often are low in nutritive value [6,7], and are characterized by low protein, low energy density and high bulk density. Cereals constitute the primary basis for most of the traditional weaning foods in Africa.

In this research, we investigated the use of processed chickpea for producing infant follow on formula without compromising the nutrition quality.

Chickpea was explored for this purpose because of its high nutrient composition and economic value. It is a good source of high quality protein, carbohydrates, vitamins (thiamine and niacin), minerals (calcium, phosphorous, iron, magnesium, and potassium) and its oil is rich with the essential fatty acid, linoleic [8,9]. Chickpea protein quality is similar to that of soy but with chickpea out scoring soy in seven of the ten essential amino acids in quantity.

Many attempts have been made to use chickpeas in non-dairy infant formulas [10-12] and in weaning food blends [13-15]. The protein efficiency ratio (PER), net protein retention (NPR) and net protein utilization (NPU) of chickpea based infant formula were not different to soy or milk based formula [10]. Similarly, when fed to malnourished children, there was no significant difference between chickpea based formula and commercial soy formula in the samples tested [11]. The mean percentage of absorption, retention and biological value of the chickpea formula were 72.4, 26.4 and 35.1 compared to that of soy 69.6, 24.3 and 34.0, respectively.

However, the presence of anti-nutritional factors, such as oligosaccharides, trypsin inhibitors, phytic acid, tannin, and haemagglutinin has limited application of chickpea in food industry [16]. We recently reported that germinating chickpeas for 72 hours followed by boiling, drying and dehulling results in highest reduction in anti-nutritional factors with minimal nutrient loss [17]. We hypothesized that the resulting chickpea flour can be used for weaning food/infant follow-on formula production. The effect of cooking of germinated and dehulled chickpeas on protein content and its amino acid profile is yet to be fully evaluated.

Therefore, in an attempt to use chickpea in weaning food production, we investigated the effect of boiling, drying and dehulling on protein quantity and amino acid profile of germinated chickpea seeds. In addition, we designed, formulated, and determined the nutritional quality of chickpea-based infant follow on formula.

## Materials and methods

### Materials

One *kabuli* cultivar of chickpea (*Cicer arietinum* L.), namely *bar,* was obtained from Volcani Center, Israelis Agricultural Research Institute and one *desi* cultivar of chickpea, namely *ICC14088*, was obtained from the Institute of Plant Sciences at the Hebrew University of Jerusalem. All reagents were bought from Sigma-Aldrich Israel.

### Chickpea flour processing

Chickpea grains were either (a) soaked, germinated, boiled dried and dehulled (GB) or (b) soaked, germinated, dried and dehulled (G) described by [17]. Briefly, Alcohol (70% ethanol) disinfected chickpea samples were soaked in water in the ratio 1:10 (w/v) for 12 hours and rinsed in water. The seeds were later placed in tray lined with absorbent paper and was later allowed to germinate at room temperature under dark environment. The seeds were rinsed with water at 12 hours interval and germination was terminated after 72 hours by freezing at −20°C for 12 hours. Part of the germinated chickpea seeds were dried immediately after defrosting in a conventional air drier (50°C) for 20 hours and labelled (G). The remainder of the germinated chickpea seeds were placed in boiling water for 3 minutes after defrosting and rinsed 3 times in hot water in order to get rid of the foam before boiling them. Chickpeas were cooked in boiling water (95–97°C) for 15 minutes and were immediately rinsed with hot water. The germinated chickpeas were dried in conventional air drier (50°C) for 20 hours and labelled (GB). All seeds were manually dehulled before milling using a heavy duty blender (Model RM100, Duisburg, Germany) to pass through a 200 micrometres sieve.

### Rate of starch hydrolysis

Chickpea flour was mixed with water (9% ^w^/_v_) and heated at 90°C for 15 minutes. Temperature of the mixture was later reduced to 75°C and saccharifying amylase was added. The progress of starch hydrolysis was monitored by measuring the change in maltose and glucose concentration over time at 30 minutes interval until 120 minutes.

### Production of follow-on formulae

After saccharification, corn oil, sucrose, carrageenan, vitamin premix, mineral premix and vanilla - banana flavour were added in amounts as described in Table 1. The mixture was blended at maximum speed for 3 minutes using a kitchen blender to liquefy.

**Table 1:** The proposed chickpea based infant follow-on formula formulation

**Ingredient**	**Percent (%)**	**Ingredient**	**Percent (%)**
Chickpea flour	8.26	Vitamin premix	9.95 × 10-5
Oil (corn oil)	2.44	Mineral premix	3.31 × 10-5
Water	87.58	Carrageenan	0.02
Sucrose	1.20	Vannila or Banana flavor	0.50

### General methods

Protein content was determined using AOAC (1990) method number 14.026 and amino acids were determined on HPLC using the procedure described by [8]. FAO/WHO reference amino acid pattern for 6–36 months old children was used to calculate the essential amino acid score [18].

Disaccharides (sucrose and maltose) and glucose were determined by HPLC. Chickpea flour (3 g) was mixed with 20 ml of water (85°C), 0.5 ml carrez reagent 1 and 0.5 ml carrez reagent 2. The mixture was heated at 85°C for 20 minutes in a water bath after which it was allowed to cool down. Water was later added to make it to 25 ml and centrifuged at 6000 rpm for 7 minutes. The supernatants were filtered through a 0.45 μm membrane paper and the filtrate was injected to HPLC. HPLC column (6.5 × 300 mm, HPX-87P BIO-RAD) was used at 80°C, using HPLC grade water as a mobile phase at the flow rate of 0.5 ml/minute and the injection volume of 40 μl. The running time was 18 minutes and detection temperature of 50°C. Chickpeas sugar compounds were identified by comparing the retention times of respective standards.

### Statistical analysis

Protein content analyses were conducted in triplicate and its data was analysed using one way analysis of variance on a JMP 07 statistical software (SAS Institute Inc., Cary, NC). Sample means were compared using Tukey HSD method and significant differences determined at p < 0.05.

## Results

### Effect on protein content

We used two chickpea cultivars (desi and kabuli) grown in Israel to determine the protein content. The results (Table 2) suggested that desi has less protein content compared to Kabuli in the studied cultivars. We determined the effect of (a) germination, and dehulling (G); (b) germination, cooking and dehulling (GB); (c) dehulling and boiling; and dehulling, soaking and boiling on the protein content of chickpeas. The protein content increased higher in the *desi cultivar* (25%) compared to *Bar* (12%) in all process types suggesting that the increment was largely due to dehulling. Our results showed that after processing, the protein content was not significantly different between cultivars, suggesting that protein distribution in the cotyledon is uniform regardless of chickpea cultivar type.

**Table 2:** The effect of processing on protein content

**Process**	**Protein (%)**	
	***Desi (ICCI4088)***	***Kabuli (Bar)***
Raw	16.66 ± 0.35^b2^	20.24 ± 0.50^b1^
Germinated, and dehulled (G)	21.34 ± 1.20^a1^	22.56 ± 0.95^a1^
Germinated, boiled and dehulled (GB)	20.00 ± 0.15^a2^	21.98 ± 0.80^a1^
Dehulled and boiled (DB)	20.95 ± 0.92^a1^	22.70 ± 0.53^a1^
Dehulled, soaked and boiled (DSB)	21.36 ± 0.85^a1^	22.78 ± 0.50^a1^

Data presented as means ± standard deviation (n = 3).

Means in the same column with different letter superscripts are significantly different (p < 0.05).

Means in the same row with the different number superscripts are significantly different (p < 0.05).

### Effect on amino acid profile

Amino acid profiles for G and GB chickpeas were compared. The result (Table 3) showed random but insignificant changes to the profile of amino acid following cooking. There were no changes in amino acids. The amino acid content in chickpeas was found to meet the amino acid profile of the WHO/FAO reference protein for 0.5 – 1 and 1 – 2 years children [18].

**Table 3:** The amino acid profile of chickpeas flour after processing

**Amino acid**	**G**	**GB**	**FAO/WHO reference protein**	
			**(0.5–1 yr)**	**(1–2 yr)**
Histidine	2.42	2.51	2	1.8
Isoleucine	4.16	4.03	3.2	3.1
Leucine	7.59	7.35	6.6	6.3
Lysine	6.04	6.10	5.7	5.7
Threonine	4.78	4.30	3.1	2.7
Valine	4.98	4.90	4.3	4.2
*Total aromatic amino acid*	8.60	8.44	5.2	4.6
*Total sulphur amino acids*	4.64	4.74	2.8	2.6
Alanine	4.59	4.47		
Aspartic acid	13.34	13.24		
Cystine	2.61	2.83		
Glutamic acid	18.51	18.95		
Glycine	4.01	3.87		
Proline	4.35	4.79		
Serine	5.22	5.23		
Tyrosine	3.00	2.83		
Methionine	2.03	1.91		
Phenylanine	5.61	5.61		
Arginine	6.81	6.92		

**Note: G** stands for soaked, germinated, and dehulled and GB = soaked, germinated, boiled and dehulled. Kabuli cultivar (bar) was used.

### Development of infant follow-on formula

The starch component of the carbohydrate was hydrolyzed with saccharifying enzymes to maltose and other smaller sugars for easy digestion by infants. The level of sugars remained constant after 30 minutes suggesting that hydrolysis time should be 30 minutes (Table 4). The results of sucrose and maltose have been reported together due to poor resolution between the two sugars. Our results suggest that at least 60% of the starch had been hydrolysed.

**Table 4:** Levels of sugars during hydrolysis of starch

**Time (minutes)**	**0**	**30**	**60**	**90**	**120**
**Sucrose and maltose (%)**	0.18	2.54	2.30	2.38	2.47
**Glucose (%)**	0	0.94	0.90	0.68	0.90
**Total**	0.18	3.48	3.20	3.06	3.37

Finally, we adjusted the nutrient value of our developed product by fortification with minerals and vitamins for compliance with infant nutrition needs. The resulting formula is shown in Table 5.

**Table 5:** Estimated nutrition value of the formulated product before fortification

	**Chickpea based weaning formula**		**Requirement per 100 kcal**	
**Nutrient**	**Per 100 ml**	**Per 100 kcal**	**Minimum**	**Maximum**
Calcium, mg	14.70	23.48	50	140
Phosphorous, mg	33.22	53.07	25	90
Magnesium, mg	12.33	19.70	5	15
Manganese, mg	0.18	0.28	0.001	0.1
Iron, mg	0.39	0.62	0.6	2
Zinc, mg	0.31	0.50	0.5	1.5
Potassium, mg	23.63	37.74	60	160
Sodium, mg	4.01	6.41	20	60
Copper, mg	0.04	0.06	0.035	0.1
Protein, g	1.83	2.92	2.25	3.5
Sucrose, g	1.77	2.84		
Carbohydrates, g	4.87	7.7		
Fat, g	3.00	4.80	4	6
Total carbohydrates, g	6.64	10.61	9	14
Energy kcal	62.59	100.00	60/100 ml	70/100 ml

## Discussion

In this study we explored the possibility of utilizing the whole chickpea grain for the production of infant follow-on formula without compromising nutritive value and quality. Currently, soy is used in both weaning and infant formulas. Chickpea protein quality is comparable to soy, however the cost of chickpea protein isolate would be much higher, as soy contains oil as a secondary high value product. The presence of antinutritional factors also limited its application in infant formulas.

We previously reported that germination for 72 hours followed by boiling, drying, and dehulling [GB] comparably resulted in chickpea flour with less antinutritional factors content than all the other investigated processes in our research [17]. Chickpea protein content range between 18 to 24% and might increase to 24 or 28% after dehulling or germination, respectively [16,19-23]. Chickpeas have been shown to vary greatly in their nutrient distribution with cultivar [24-27]. The two broad categories of chickpea (desi or kabuli) were used in our study. First it was important to note that on average the protein content of chickpeas was not different after dehulling regardless of cultivar type meaning that any cultivar can be used. Second, boiling of germinated grains followed by dehulling does not affect negatively the amino acid profile of chickpea proteins.

Conventionally, novel infant follow on formulas are exclusively made from either cow’s milk protein or soy protein isolate which is blended with starch or corn syrup or dextrose, vegetable oil/fat and mineral and vitamin premixes among other ingredients [5]. The ratio of protein and carbohydrate in chickpea proximate composition is 0.25, which is similar to that of an infant follow-on formula formulation, suggesting that the whole chickpea grain can be utilized [8,16]. Therefore, we used chickpea as a common source for protein, carbohydrate and minerals with minimal supplementation of the later. The infant follow-on formula was formulated to meet the requirements stipulated in the codex standard on infant follow-on formula and EC directive [28,29]. The estimated nutrition value of developed chickpea based infant follow-on formula without fortification (Table 5) showed the need for calcium, sodium, and potassium supplementation to meet the requirements. Furthermore, we assumed loss of significant amount of water-soluble vitamins such that vitamin supplementation should meet the daily infant nutrition needs.

The process thereof is as follows; chickpea flour from process [GB] is mixed with boiling water. The mixture is kept at 90°C for 15 minutes and saccharifying enzymes are added after the temperature reduces to 75–80°C to hydrolyse starch for 30 minutes. The volume is adjusted to initial volume by adding water. Corn oil, sucrose, carrageenan, vitamin premix, mineral premix and flavour are added to the mixture which is later blended at maximum speed for 3 minutes. The product will later undergo UHT treatment and aseptically packed to ensure safety to children.

The developed product meets the minimum nutrition requirements of codex alimenterious and those of EU regulations. Also, literature indicates that chickpeas true protein digestibility, biological value, net protein utilization and protein efficiency ratio is higher than most legumes including soybean [11,12,30,31]. Since the whole chickpea is used, the cost of production is lower than existing formula products on the market. Therefore, the developed product does have the potential to be used in developing countries without compromising the nutrition quality. However, clinical trial might be needed to access the efficacy of the product.

The developed infant follow-on formula will not only benefit developing countries but also developed countries as an alternative to or replacement of soy based formulas. Unlike chickpea, soy contains phytoestrogens which have been reported to initiate estrogen receptor-mediated reactions as reviewed by [32,33]. Evidence from animal studies suggests that phytoestrogens have adverse effect on reproductive system even though not yet significantly correlated in human studies [34,35]. In human studies, ingestion of isoflavones rich diet by premenopausal women resulted in increased menstrual cycle [36]. In a retro respective cohort study done by Iowa University, it was found that women fed on soy based formula during infancy had a prolonged menstrual bleeding and felt greater discomfort with menstruation than those fed on cow’s milk based formula [37]. A recent study in Israel with 694 infants aged between 3–24 months indicated that soy based formulas slow infantile breast tissue wearing [38]. Consequently, different governments have cautioned the general public from unnecessarily exposing infants to soy formula.

## Conclusion

As shown in this study, chickpea can be used for infant weaning food/follow-on formula with minimal mineral and vitamin fortification. Germination for 72 hours followed by boiling, drying and dehulling increases the protein content but does not affect the amino acid profile of chickpeas. Similarly, we have shown that the difference in protein content of the seeds between cultivars is not important as final concentration of protein after processing does not vary with cultivar. The formulated chickpea based follow-on formula prior to micronutrient supplementation meets almost all the nutrient requirements of EC directive on infant follow-on formula except for calcium, sodium, and potassium.

This work will hopefully further encourage the introduction of chickpea based foods in places where this legume is part of the local staple food.

## Competing interests

The authors declare that they have no competing interests.

## Authors’ contributions

LNM- participated in study design and writing the manuscript, and conducted the experiments. SBED- participated in experiments and drafting the manuscript. EZ- provided technical assistance. ZB *–* provided technical support. SA- plant geneticist. RR- head of group. All authors read and approved the final manuscript.
